# High Virulence and Antifungal Resistance in Clinical Strains of* Candida albicans*

**DOI:** 10.1155/2016/5930489

**Published:** 2016-12-12

**Authors:** Eric Monroy-Pérez, Gloria Luz Paniagua-Contreras, Pamela Rodríguez-Purata, Felipe Vaca-Paniagua, Marco Vázquez-Villaseñor, Clara Díaz-Velásquez, Alina Uribe-García, Sergio Vaca

**Affiliations:** Facultad de Estudios Superiores Iztacala, Universidad Nacional Autónoma de México, Av. de los Barrios 1, Los Reyes Iztacala, 54090 Tlalnepantla, MEX, Mexico

## Abstract

Antifungal resistance and virulence properties of* Candida albicans *are a growing health problem worldwide. To study the expression of virulence and azole resistance genes in 39 clinical strains of* C. albicans*, we used a model of infection of human vaginal epithelial cells with* C. albicans *strains isolated from Mexican women with vulvovaginal candidiasis (VVC). The strains were identified by PCR amplification of the ITS1 and ITS2 regions of rRNA. The detection and expression of virulence genes and azole resistance genes* MDR1* and* CDR1* were performed using PCR and RT-PCR, respectively. All strains were sensitive to nystatin and 38 (97.4%) and 37 (94.9%) were resistant to ketoconazole and fluconazole, respectively.* ALS1*,* SAP4–SAP6*,* LIP1*,* LIP2*,* LIP4*,* LIP6*,* LIP7*,* LIP9*,* LIP10*, and* PLB1*-*PLB2* were present in all strains;* SAP1* was identified in 37 (94.8%) isolates,* HWP1* in 35 (89.7%),* ALS3* in 14 (35.8%), and* CDR1* in 26 (66.6%). In nearly all of the strains,* ALS1*,* HWP1*,* SAP4–SAP6*,* LIP1–LIP10*,* PLB1*, and* PLB2* were expressed, whereas* CDR1* was expressed in 20 (51.3%) and* ALS3* in 14 (35.8%). In our in vitro model of infection with* C. albicans*, the clinical strains showed different expression profiles of virulence genes in association with the azole resistance gene* CDR1*. The results indicate that the strains that infect Mexican patients suffering from VVC are highly virulent and virtually all are insensitive to azoles.

## 1. Introduction

*Candida albicans* is an opportunistic fungus that infects the mucosae of oral and vaginal cavities [1]. Vulvovaginal candidiasis (VVC) is among the most common infections that affect women of childbearing age [2]. Clinical strains of* C. albicans* possess an array of virulence genes that directly influence the pathogenesis of VVC, including genes responsible for phenotypic switching [3],* HWP1* (hyphal wall protein 1) [4],* ALS* (agglutinin-like sequence) [5],* SAP* (secreted aspartyl proteases) [6],* PL* (phospholipases) [7], and* LIP* (lipases) [8]. The aspartyl proteases, phospholipases, and lipases are involved in the breakdown of cell membranes in host epithelia, which promotes colonization and invasion [9].

The increase of* C. albicans* resistant to antifungal agents, mainly to azoles, is a serious health problem and hampers the treatment of VVC. The mechanisms responsible for resistance to azoles include overexpression of* CDR1* and* CDR2* from the ABC transporter family and* MDR1* which encodes a multidrug efflux pump. Mutations in* ERG5* and* ERG11*, which cause alterations in C22-desaturase and sterol 14*α*-demethylase, respectively, are also associated with azole resistance [10].

The expression of* ALS* and* SAP* gene families in* C. albicans* of vaginal origin has recently been studied [11, 12]. Likewise, the gene expression patterns of the* LIP* and* PL* family, which are associated with the formation of biofilm during infection, have been studied [13]. However, the expression patterns of* ALS*,* HWP1*,* SAP*,* LIP*, and* PLB* in association with azole resistance genetic determinants have been less studied in strains of vaginal origin. In this work, to analyse the expression patterns of* ALS1–ALS3*,* HWP1*,* SAP1*,* SAP4–SAP6*,* LIP1–LIP10*,* PLB1*-*PLB2*,* CDR1*, and* MDR1*, we implemented an in vitro model of infection of human vaginal cells with* C. albicans* strains isolated from women with VVC.

## 2. Materials and Methods

### 2.1. Patients Analysed and Sampling

Our study included a group of 200 women (ages 18–57) who attended the gynaecology department of public hospitals in the State of Mexico, Mexico, and presented characteristic symptoms of VVC (itching, burning, dysuria, and curd-like discharge). In this study, adult women with/without active sexual life were included. The exclusion criteria were as follows: patients with cervical cancer, being pregnant, being under antibiotic treatment, or having ingested antifungal drugs in the last 30 days. In addition, the patients informed if they had been previously exposed to azoles at least once in the last year as treatment for VVC. All participating women signed a letter of informed consent. The ethics committee of each hospital approved the study. Two samples were taken from the cervicovaginal cavity of each patient, using sterile swabs. The first was used to observe the presence of yeast, hyphae, or pseudohyphae by light microscopy. The second was incubated for 24 h at 37°C in brain heart infusion (BHI) medium (BD Bioxon, Cuautitlan Izcalli, State of Mexico, Mexico). After 24 h, the samples were incubated at 37°C in Sabouraud agar plates (BD Bioxon) with 50 *μ*g/mL chloramphenicol.

### 2.2. Identification of* C. albicans*

*C. albicans* strains were identified by the API 20 C Aux test (bioMerieux, Durham, NC, USA), germ tube test [11], and PCR amplification of ITS1 and ITS2 rRNA loci (the primers are shown in Table 1) [14]. The DNA of the strains was extracted by boiling, as described by Paniagua-Contreras et al. [15]. The* C. albicans* strain B311 (ATCC 32354, ATCC, Manassas, VA, USA) was used as a positive control.

### 2.3. Antifungal Susceptibility

To test* C. albicans* antifungal susceptibility, we used diffusion disks impregnated with 25 *μ*g of fluconazole, 10 *μ*g of ketoconazole, and 100 U nystatin (HiMedia Laboratories, Mumbai, India). Antifungal susceptibility was established according to the interpretation criteria previously described (Table 2) [16].

### 2.4. Detection of Genetic Virulence Markers and Azole Resistance Genes by PCR

The primers and PCR conditions used were previously described: ALS1 and ALS3 [17], HWP1 [18], SAP1 [19], SAP4–SAP6 [20], LIP1–LIP10 and PLB1-PLB2 [13], and CDR1 and MDR1 [21] (Table 1).

### 2.5. In Vitro Infection Model

To study the expression of virulence markers and azole resistance genes, we used the A431 cell line (ATCC CRL-1555), which is derived from a vulvar epidermoid carcinoma. The growth conditions of the strains were previously reported [22, 23]. The A431 cell culture was inoculated with 50 *μ*L of phosphate-buffered saline (PBS) with 2 × 10^6^ *C. albicans *cells. The mixed cultures were incubated for 72 h at 37°C in 5% CO_2_. The culture medium was changed every 24 h.* C. albicans* (ATCC 32354) and* Staphylococcus epidermidis* (ATCC 35984) were used as positive and negative controls, respectively.

### 2.6. RNA Purification and cDNA Synthesis

After infection of A431 cells, the yeasts were harvested and suspended in 200 *μ*L of buffer Y1 (Qiagen, Hilden, Germany) with lyticase (50 U/10^7^ cells). Samples were incubated for 20 min at 30°C with gentle agitation to facilitate the formation of spheroplasts. Total RNA was purified on a robotic QIAcube workstation (Qiagen) using the RNeasy Mini Kit (Qiagen), following the manufacturer's instructions, including DNase treatment. The concentration and purity of total RNA were analysed on a NanoDrop spectrophotometer 2000 (Thermo Scientific, Waltham, MA, USA). cDNA was synthesized with the QuantiTect Reverse Transcription kit (Qiagen) according to manufacturer's instructions.

### 2.7. Amplification and Detection of the Azole Resistance and Virulence Genes

Amplification and detection of azole resistance and virulence genes were performed using real-time PCR with the Rotor-Gene SYBR Green PCR kit (Qiagen). The primer sequences used for the gene expression analysis are shown in Table 1. The final volume of each reaction was 25 *μ*L, consisting of 12.5 *μ*L of SYBR Green Master Mix, 1 *μ*L of forward primer (1 *μ*M), 1 *μ*L of reverse primer (1 *μ*M), 2 *μ*L of cDNA (~20 ng), and 8.5 *μ*L RNase-free water. Amplification conditions were 95°C for 5 min, 40 cycles at 95°C for 5 s, and combination alignment/extension at 60°C for 10 s. The amplification conditions for the positive and negative controls were the same as described above.

## 3. Results

### 3.1. Identification of* C. albicans* and Antifungal Resistance Phenotypes

*C. albicans* was identified in 19.5% (*n* = 39) of the samples and, in the remaining ones (80.5%, *n* = 161), bacteria such as* Escherichia coli*,* Klebsiella *spp.,* Staphylococcus aureus*, or* Staphylococcus epidermidis* were found.

All 39 isolates were sensitive to nystatin, whereas 97.4% (*n* = 38) and 94.9% (*n* = 37) were resistant to fluconazole and ketoconazole, respectively (Table 3). Resistance to both azoles was found in 94.9% (*n* = 37) of the strains.

### 3.2. Detection of Virulence Markers and Azole Resistance Genes

All of the isolated strains (*n* = 39) were carriers of the* ALS1, SAP4*-*SAP6*,* LIP1*,* LIP2*,* LIP4*,* LIP6*,* LIP7*,* LIP9*,* LIP10,* and* PLB1*–*PLB2 *genes. Other genes detected were* SAP1* in 94.8% (*n* = 37),* HWP1* (89.7%, *n* = 35),* ALS3* (35.8%, *n* = 14), and* CDR1* (66.6%, *n* = 26) of the strains (Table 4).

### 3.3. Expression Patterns of the Virulence Genes and* CDR1*

In nearly all of the isolates,* ALS1*,* HWP1*,* SAP4*–*SAP6*,* LIP1*–*LIP10*,* PLB1*, and* PLB2* were expressed after infection of the cell line A431, whereas* CDR1* was expressed in 20 (51.3%) and* ALS3* in 14 (35.8%) (Table 4).

Fourteen distinct expression patterns of* ALS*,* HWP1*,* SAP*,* PLB*,* LIP*, and* CDR1* were identified (Table 5). The most prevalent expression profile, Pattern 1, composed of 18 genes, was found in 25.6% (*n* = 10) of the strains. Pattern 2 included 20 expressed genes and was found in 18% (*n* = 7); Patterns 3 and 4, both expressing 19 genes, were detected in 12.8% (5 strains); Patterns 5 (18 genes) and 6 (19 genes) were detected in 5.1% (*n* = 2) (Table 5). The following eight expression patterns (Patterns 7–14) were represented by a single strain.* CDR1* was found in eight different patterns (*n* = 20).

## 4. Discussion

In this study, we isolated and identified the virulence gene expression patterns of* C. albicans* strains in 19.5% of 200 vaginal samples. VVC is a condition that affects a quarter of young women [2, 24]. The presence of* E. coli*,* Klebsiella *spp.,* S. aureus*, or* S. epidermidis *in most vaginal samples (*n* = 161) agrees with previous reports showing bacterial vaginitis as the first cause of vaginal infections [24].

The chronicity of VVC or recurrent episodes by* C. albicans* is due to prolonged use of antifungals, which select for resistant strains, and due to the numerous virulence genotypes that promote adhesion to epithelial cells, production of hydrolytic enzymes, colonization, and invasion [9]. We found that each strain isolated that carried* ALS1* (39/39) and* ALS3* (14/14) was able to express these genes in the in vitro model of infection (Table 4). The frequency of expression of the* ALS* genes reported here is similar to those reported in vaginal candidiasis models [25]. The ALS proteins are extracellular components of the cell membrane which mediate adhesion and colonization of host cells [26, 27]. Widespread expression of these proteins in the isolated strains supports their role in infection.

All strains positive for* HWP1* (35/35) expressed this gene during infection of A431 cells (Table 4). The frequency of expression is higher than results reported in* C. albicans* strains isolated from women with vaginal infection in Turkey [28]. The high frequency of expression of* HWP1 *and the* ALS* genes found in this study (Table 4) reveals the capacity of the isolated strains to cause chronic vaginal infections. This result is consistent with reports showing that the combined expression of the adhesion proteins HWP1 and ALS1–ALS3 significantly facilitates biofilm formation [29] and can participate in the resistance to antifungals by limiting their penetrance to the biofilm [30].

Likewise, all isolated strains expressed* SAP4*–*SAP6* (39/39) and almost all expressed* SAP1* (92.3%, (36/37)) (Table 4). These results are similar to those previously described for* SAP* expression in VVC in humans [31]. The expression of* SAP4*–*SAP6* has a relevant role in pathogenesis by promoting hyphae formation [32] and by the inhibition of phagocytosis [33].

In this study, most strains expressed genes of the* LIP* family, except* LIP3*,* LIP5*, and* LIP8* (Table 4). These data are consistent with those reported using an in vitro infection model of oral epithelial cells and in patient samples [34]. The specific role of LIP proteins in infection is unknown. However, it has been described that* LIP* family members are differentially expressed during infection in specific areas of affected mucosae [35].

All strains of* C. albicans* expressed* PLB1* and* PLB2* (39/39) (Table 4). Recently, phospholipase activity was reported in strains of* C. albicans* and in nonalbicans strains from patients with vulvovaginal infections [36], as well as in different* Candida* species isolated from patients with type 2 diabetes mellitus suffering from VVC [37]. The elevated expression of phospholipase genes in the* C. albicans* strains isolated in this study (Table 4) suggests that these genes are essential for vaginal infection by participating in the breakdown of the cell membrane, which allows the hyphae to penetrate the epithelial cell cytoplasm [7].

The use of azoles for the treatment of VVC over time has resulted in the selection of strains resistant to these compounds [38]. In this study, the association of azole resistance phenotypes (fluconazole/ketoconazole) was identified in 94.9% (*n* = 37) of the strains (Table 3), whereas* CDR1* was found in only 66.6% (*n* = 26) (Table 4). This gap in detection can be explained by the idea that azole resistance is not only conferred by* CDR1*, an ABC transporter, but may also be caused by the overproduction of an azole target, 14*α*-demethylase, which is encoded by* ERG11*. Other mechanisms of azole resistance are caused by mutations in* ERG11* which decrease azole affinity to this enzyme [10, 39].

Interestingly, all strains described in this study were sensitive to nystatin (*n* = 39, Table 3), as also reported in other studies [40, 41]. This result suggests that nystatin can be an alternative for the treatment of vaginal infections caused by strains of* C. albicans* which are resistant to azoles [42]. The high frequencies of strains resistant to fluconazole and ketoconazole (Table 3) can be explained by the high use of these two drugs (and clotrimazole) in Mexico, given that this is the most commonly used therapy against VVC. Our results are consistent with the observation that* Candida* species isolated in different geographical regions differ in their sensitivity to fluconazole [43].

In this study, we identified 14 distinct expression patterns of* ALS*,* HWP1*,* SAP*,* LIP*,* PLB*, and* CDR1* in A431 cells infected in vitro with strains of* C. albicans* (Table 5). Pattern 1, comprising 18 genes, was expressed by most strains (25.6%). These findings show that, during the pathogenesis of infection, most virulence markers are expressed (in 8 of the 14 patterns). The combined expression of the azole resistance gene* CDR1* with the virulence markers indicates that these strains are highly virulent and should be treated with a nonazole therapy.

## 5. Conclusion

This is the first work done in Mexico on global expression of different markers of virulence and resistance to azoles in clinical strains of* C. albicans*. The results show that the strains that infect Mexican patients suffering from VVC are highly virulent and virtually all are insensitive to azoles.

## Figures and Tables

**Table 1:** Primers used in PCR and real-time PCR assays.

Gene	Orientation	Sequence 5′ to 3′
rRNA	FW	TTTATCAACTTGTCACACCAGA
	RV	ATCCCGCCTTACCACTACCG

*ALS1*	FW	CCATCACTGAAGATATCACCACA
	RV	TGGAGCTTCTGTAGGACTGGTT

*ALS3*	FW	CCAAGTGTTCCAACAACTGAAA
	RV	GAACCGGTTGTTGCTATGGT

*HWP1*	FW	CCATGTGATGATTACCCACA
	RV	GCTGGAACAGAAGATTCAGG

*SAP1*	FW	TCAATCAATTTACTCTTCCATTTCTAACA
	RV	CCAGTAGCATTAACAGGAGTTTTAATGACA

*SAP4*	FW	TTATTTTTAGATATTGAGCCCACAGAAA
	RV	GCCAGTGTCAACAATAACGCTAAGTT

*SAP5*	FW	AGAATTTCCCGTCGATGAGACTGG
	RV	CAAATTTTGGGAAGTGCGGGAAGA

*SAP6*	FW	CCCGTTTTGAAATTAAATATGCTGATGG
	RV	GTCGTAAGGAGTTCTGGTAGCTTCG

* LIP1*	FW	AGCCCAACCAGAAGCTAATGAA
	RV	TGATGCAAAAGTCGCCATGT

*LIP2*	FW	GGCCTGGATTGATGCAAGAT
	RV	TTGTGTGCAGACATCCTTGGA

*LIP3*	FW	TCTCACCGAGATTGTTGTTGGA
	RV	GTTGGCCATCAAATCTTGCA

*LIP4*	FW	GCGTCCTGTTGCTTTCACT
	RV	ACACGGTTTGTTTTCCATTGAA

*LIP5*	FW	TGGTTCCAAAAATACCGTGTT
	RV	CGACAATAGGGACGATTTGATCA

*LIP6*	FW	AAGAATCTTCCGACCTGACCAA
	RV	ATATGCACCTGTTGACGTTCAAA

*LIP7*	FW	AACTGATATTTGCCATGCATTAGAAA
	RV	CCATTCCCGGTAACTAGCATGT

*LIP8*	FW	CAACAATTGCTAAAATCGTTGAAGA
	RV	AGGGATTTTTGGCACTAATTGTTT

*LIP9*	FW	CGCAAGTTTGAAGTCAGGAAAA
	RV	CCCACATTACAATTTGGCATCT

*LIP10*	FW	CACCTGGCTTAGCAGTTGCA
	RV	CCCAGCAAAGACTCATTTTATTCA

*PLB1*	FW	GGTGGAGAAAGATGGCCAAAA
	RV	AGCACTTACGTTACGATGCAACA

*PLB2*	FW	TGAAACCTTTGGGCGACAACT
	RV	GCCGCGCTCGTTGTTAA

*CDR1*	FW	AAGAGAACCATTACCAGG
	RV	AGGAATCGACGGATCAC

*MDR1*	FW	GGAGTTTAGGTGCTGT
	RV	CGGTGATGGCTCTCAA

**Table 2:** Interpretive criteria of susceptibility and resistance to antifungals used in this study.

Antifungal drugs	Zone diameter (mm)		
	Sensitive	Dose-dependent	Resistant
Ketoconazole	≥30	23–29	≤22
Fluconazole	≥19	15–18	≤14
Nystatin	≥25	17–24	≤14

**Table 3:** Susceptibility and resistance of *Candida albicans* strains to antifungal drugs.

Antifungal drugs	Resistant Number (%)	Sensitive Number (%)
Nystatin	0 (0)	39 (100)
Fluconazole	37 (94.9)	2 (5.1)
Ketoconazole	38 (97.4)	1 (2.6)

**Table 4:** Percentages of detection and expression of virulence markers and azole resistance genes in *Candida albicans* strains.

Gene	Detection Number (%)	Expression Number (%)
*ALS1*	39 (100)	39 (100)
*ALS3*	14 (35.8)	14 (35.8)
*HWP1*	35 (89.7)	35 (89.7)
*SAP1*	37 (94.8)	36 (92.3)
*SAP4*	39 (100)	39 (100)
*SAP5*	39 (100)	39 (100)
*SAP6*	39 (100)	39 (100)
*LIP1*	39 (100)	39 (100)
*LIP2*	39 (100)	39 (100)
*LIP3*	38 (97.4)	38 (97.4)
*LIP4*	39 (100)	39 (100)
*LIP5*	35 (89.7)	35 (89.7)
*LIP6*	39 (100)	39 (100)
*LIP7*	39 (100)	39 (100)
*LIP8*	38 (97.4)	38 (97.4)
*LIP9*	39 (100)	39 (100)
*LIP10*	39 (100)	39 (100)
*PLB1*	39 (100)	39 (100)
*PLB2*	39 (100)	39 (100)
*CDR1*	26 (66.6)	20 (51.3)
*MDR1*	0 (0)	0 (0)

**Table 5:** Expression patterns of virulence genes and *CDR1* in *Candida albicans* strains.

Pattern number	Pattern	Number of strains	%
1	*ALS1/HWP1/SAP1/SAP4/SAP5/SAP6/LIP1/LIP2/LIP3/LIP4/LIP5/LIP6/LIP7/LIP8/LIP9/LIP10/PLB1/PLB2*	10	25.6
2	*ALS1/ALS3/HWP1/SAP1/SAP4/SAP5/SAP6/LIP1/LIP2/LIP3/LIP4/LIP5/LIP6/LIP7/LIP8/LIP9/LIP10/PLB1/PLB2/CDR1*	7	18.0
3	*ALS1/ALS3/HWP1/SAP1/SAP4/SAP5/SAP6/LIP1/LIP2/LIP3/LIP4/LIP5/LIP6/LIP7/LIP8/LIP9/LIP10/PLB1/PLB2*	5	12.8
4	*ALS1/HWP1/SAP1/SAP4/SAP5/SAP6/LIP1/LIP2/LIP3/LIP4/LIP5/LIP6/LIP7/LIP8/LIP9/LIP10/PLB1/PLB2/CDR1*	5	12.8
5	*ALS1/SAP1/SAP4/SAP5/SAP6/LIP1/LIP2/LIP3/LIP4/LIP5/LIP6/LIP7/LIP8/LIP9/LIP10/PLB1/PLB2/CDR1*	2	5.1
6	*ALS1/HWP1/SAP1/SAP4/SAP5/SAP6/LIP1/LIP2/LIP3/LIP4/LIP5/LIP6/LIP7/LIP8/LIP9/LIP10/PLB1/PLB2/CDR1*	2	5.1
7	*ALS1/HWP1/SAP4/SAP5/SAP6/LIP1/LIP2/LIP3/LIP4/LIP6/LIP7/LIP8/LIP9/LIP10/PLB1/PLB2*	1	2.5
8	*ALS1/ALS3/HWP1/SAP1/SAP4/SAP5/SAP6/LIP1/LIP2/LIP3/LIP4/LIP6/LIP7/LIP8/LIP9/LIP10/PLB1/PLB2*	1	2.5
9	*ALS1/SAP1/SAP4/SAP5/SAP6/LIP1/LIP2/LIP3/LIP4/LIP5/LIP6/LIP7/LIP8/LIP9/LIP10/PLB1/PLB2*	1	2.5
10	*ALS1/HWP1/SAP1/SAP4/SAP5/SAP6/LIP1/LIP2/LIP3/LIP4/LIP6/LIP7/LIP8/LIP9/LIP10/PLB1/PLB2/CDR1*	1	2.5
11	*ALS1/HWP1/SAP1/SAP4/SAP5/SAP6/LIP1/LIP2/LIP3/LIP4/LIP6/LIP7/LIP9/LIP10/PLB1/PLB2*	1	2.5
12	*ALS1/HWP1/SAP1/SAP4/SAP5/SAP6/LIP1/LIP2/LIP3/LIP4/LIP5/LIP6/LIP7/LIP8/LIP9/LIP10/PLB1/PLB2/CDR1*	1	2.5
13	*ALS1/ALS3/HWP1/SAP4/SAP5/SAP6/LIP1/LIP2/LIP3/LIP4/LIP5/LIP6/LIP7/LIP8/LIP9/LIP10/PLB1/PLB2/CDR1*	1	2.5
14	*ALS1/HWP1/SAP4/SAP5/SAP6/LIP1/LIP2/LIP3/LIP4/LIP5/LIP6/LIP7/LIP8/LIP9/LIP10/PLB1/PLB2/CDR1*	1	2.5

## References

[1] Odds F. C., Gow N. A., Brown A. J., Heitman J., Filler S. G., Edwards J. E. Jr., Mitchell A. P. (2006). Toward a molecular understanding of *Candida albicans* virulence. *Molecular Principles of Fungal Pathogenicity*.

[2] Sobel J. D., Faro S., Force R. W. (1998). Vulvovaginal candidiasis: epidemiologic, diagnostic, and therapeutic considerations. *American Journal of Obstetrics and Gynecology*.

[3] Hellstein J., Vawter-Hugart H., Fotos P., Schmid J., Soll D. R. (1993). Genetic similarity and phenotypic diversity of commensal and pathogenic strains of Candida albicans isolated from the oral cavity. *Journal of Clinical Microbiology*.

[4] Sobel J. D., Muller G., Buckley H. R. (1984). Critical role of germ tube formation in the pathogenesis of candidal vaginitis. *Infection and Immunity*.

[5] Zhao X., Oh S.-H., Cheng G. (2004). ALS3 and ALS8 represent a single locus that encodes a Candida albicans adhesin; functional comparisons between Als3p and Als1p. *Microbiology*.

[6] Hube B. (1998). Possible role of secreted proteinases in Candida albicans infections. *Revista Iberoamericana de Micologia*.

[7] Ghannoum M. A. (2000). Potential role of phospholipases in virulence and fungal pathogenesis. *Clinical Microbiology Reviews*.

[8] Hube B., Stehr F., Bossenz M., Mazur A., Kretschmar M., Schäfer W. (2000). Secreted lipases of Candida albicans: cloning, characterisation and expression analysis of a new gene family with at least ten members. *Archives of Microbiology*.

[9] Schaller M., Borelli C., Korting H. C., Hube B. (2005). Hydrolytic enzymes as virulence factors of Candida albicans. *Mycoses*.

[10] Manastir L., Ergon M. C., Yücesoy M. (2011). Investigation of mutations in Erg11 gene of fluconazole resistant Candida albicans isolates from Turkish hospitals. *Mycoses*.

[11] Monroy-Pérez E., Sáinz-Espuñes T., Paniagua-Contreras G., Negrete-Abascal E., Rodríguez-Moctezuma J. R., Vaca S. (2012). Frequency and expression of *ALS* and *HWP1* genotypes in *Candida albicans* strains isolated from Mexican patients suffering from vaginal candidosis. *Mycoses*.

[12] Monroy-Pérez E., Paniagua-Contreras G., Vaca-Paniagua F., Negrete-Abascal E., Vaca S. (2013). SAP expression in *Candida albicans* strains isolated from Mexican patients with vaginal candidosis. *International Journal of Clinical Medicine*.

[13] Nailis H., Kucharíkov S., Řičicová M. (2010). Real-time PCR expression profiling of genes encoding potential virulence factors in *Candida albicans* biofilms: identification of model-dependent and -independent gene expression. *BMC Microbiology*.

[14] Luo G., Mitchell T. G. (2002). Rapid identification of pathogenic fungi directly from cultures by using multiplex PCR. *Journal of Clinical Microbiology*.

[15] Paniagua-Contreras G. L., Monroy-Pérez E., Rodríguez-Moctezuma J. R., Domínguez-Trejo P., Vaca-Paniagua F., Vaca S. (2015). Virulence factors, antibiotic resistance phenotypes and O-serogroups of Escherichia coli strains isolated from community-acquired urinary tract infection patients in Mexico. *Journal of Microbiology, Immunology and Infection*.

[16] Pfaller M. A., Diekema D. J., Colombo A. L. (2006). Candida rugosa, an emerging fungal pathogen with resistance to azoles: geographic and temporal trends from the ARTEMIS DISK Antifungal Surveillance Program. *Journal of Clinical Microbiology*.

[17] Green C. B., Cheng G., Chandra J., Mukherjee P., Ghannoum M. A., Hoyer L. L. (2004). RT-PCR detection of Candida albicans ALS gene expression in the reconstituted human epithelium (RHE) model of oral candidiasis and in model biofilms. *Microbiology*.

[18] Naglik J. R., Fostira F., Ruprai J., Staab J. F., Challacombe S. J., Sundstrom P. (2006). Candida albicans HWP1 gene expression and host antibody responses in colonization and disease. *Journal of Medical Microbiology*.

[19] Hube B., Turver C. J., Odds F. C. (1991). Sequence of the *Candida albicans* gene encoding the secretory aspartate proteinase. *Journal of Medical and Veterinary Mycology*.

[20] Naglik J. R., Rodgers C. A., Shirlaw P. J. (2003). Differential expression of *Candida albicans* secreted aspartyl proteinase and phospholipase B genes in humans correlates with active oral and vaginal infections. *Journal of Infectious Diseases*.

[21] Lee M.-K., Williams L. E., Warnock D. W., Arthington-Skaggs B. A. (2004). Drug resistance genes and trailing growth in Candida albicans isolates. *Journal of Antimicrobial Chemotherapy*.

[22] Schaller M., Korting H. C., Schäfer W., Bastert J., Chen W., Hube B. (1999). Secreted aspartic proteinase (Sap) activity contributes to tissue damage in a model of human oral candidosis. *Molecular Microbiology*.

[23] Schaller M., Schäfer W., Korting H. C., Hube B. (1998). Differential expression of secreted aspartyl proteinases in a model of human oral candidosis and in patient samples from the oral cavity. *Molecular Microbiology*.

[24] Sobel J. D. (1997). Vaginitis. *New England Journal of Medicine*.

[25] Cheng G., Wozniak K., Wallig M. A., Fidel P. L., Trupin S. R., Hoyer L. L. (2005). Comparison between Candida albicans agglutinin-like sequence gene expression patterns in human clinical specimens and models of vaginal candidiasis. *Infection and Immunity*.

[26] Hoyer L. L., Clevenger J., Hecht J. E., Ehrhart E. J., Poulet F. M. (1999). Detection of Als proteins on the cell wall of Candida albicans in murine tissues. *Infection and Immunity*.

[27] Hoyer L. L., Payne T. L., Hecht J. E. (1998). Identification of Candida albicans ALS2 and ALS4 and localization of Als proteins to the fungal cell surface. *Journal of Bacteriology*.

[28] Nas T., Kalkanci A., Fidan I. (2008). Expression of ALS1, HWP1 and SAP4 genes in Candida albicans strains isolated from women with vaginitis. *Folia microbiologica*.

[29] Nobile C. J., Schneider H. A., Nett J. E. (2008). Complementary Adhesin Function in C. albicans Biofilm Formation. *Current Biology*.

[30] Douglas L. J. (2003). Candida biofilms and their role in infection. *Trends in Microbiology*.

[31] Lian C. H., Liu W. D. (2007). Differential expression of *Candida albicans* secreted aspartyl proteinase in human vulvovaginal candidiasis. *Mycoses*.

[32] Newport G., Agabian N. (1997). KEX2 influences Candida albicans proteinase secretion and hyphal formation. *Journal of Biological Chemistry*.

[33] Borg-von Zepelin M., Beggah S., Boggian K., Sanglard D., Monod M. (1998). The expression of the secreted aspartyl proteinases Sap4 to Sap6 from *Candida albicans* in murine macrophages. *Molecular Microbiology*.

[34] Stehr F., Felk A., Gácser A. (2004). Expression analysis of the Candida albicans lipase gene family during experimental infections and in patient samples. *FEMS Yeast Research*.

[35] Schofield D. A., Westwater C., Warner T., Balish E. (2005). Differential Candida albicans lipase gene expression during alimentary tract colonization and infection. *FEMS Microbiology Letters*.

[36] Fule S. R., Das D., Fule R. P. (2015). Detection of phospholipase activity of *Candida albicans* and non albicans isolated from women of reproductive age with vulvovaginal candidiasis in rural area. *Indian Journal of Medical Microbiology*.

[37] Bassyouni R. H., Wegdan A. A., Abdelmoneim A., Said W., Aboelnaga F. (2015). Phospholipase and aspartyl proteinase activities of candida species causing vulvovaginal candidiasis in patients with type 2 diabetes mellitus. *Journal of Microbiology and Biotechnology*.

[38] Richter S. S., Galask R. P., Messer S. A., Hollis R. J., Diekema D. J., Pfaller M. A. (2005). Antifungal susceptibilities of Candida species causing vulvovaginitis and epidemiology of recurrent cases. *Journal of Clinical Microbiology*.

[39] Alvarez-Rueda N., Fleury A., Logé C. (2016). The amino acid substitution N136Y in *Candida albicans* sterol 14 *α*-demethylase is involved in fluconazole resistance. *Medical Mycology*.

[40] Wang F. J., Zhang D., Liu Z. H., Wu W. X., Bai H. H., Dong H. Y. (2016). Species distribution and in vitro antifungal susceptibility of vulvovaginal Candida isolates in China. *Chinese Medical Journal*.

[41] Choukri F., Benderdouche M., Sednaoui P. (2014). In vitro susceptibility profile of 200 recent clinical isolates of *Candida* spp. to topical antifungal treatments of vulvovaginal candidiasis, the imidazoles and nystatin agents. *Journal de Mycologie Médicale*.

[42] Achkar J. M., Fries B. C. (2010). Candida infections of the genitourinary tract. *Clinical Microbiology Reviews*.

[43] Yang Y.-L., Cheng H.-H., Ho Y.-A., Hsiao C.-F., Lo H.-J. (2003). Fluconazole resistance rate of Candida species from different regions and hospital types in Taiwan. *Journal of Microbiology, Immunology and Infection*.

